# A Type A Aortic Dissection Mimicking an Acute Myocardial Infarction

**DOI:** 10.4021/cr151w

**Published:** 2012-03-20

**Authors:** Antonio D’Aloia, Enrico Vizzardi, Silvia Bugatti, Marco Magatelli, Ivano Bonadei, Riccardo Rovetta, Filippo Quinzani, Antonio Curnis, Livio Dei Cas

**Affiliations:** a Section of Cardiovascular and Cardiosurgery Disease, Department of Applied Experimental Medicine,Brescia Study University, Italy

**Keywords:** Aortic dissection, Myocardial infarction

## Abstract

We describe a case of a 54 years old man in whom an initial diagnosis of acute coronary syndrome (ACS) revealed to be finally an acute aortic dissection. This case report stresses the importance to maintain a high grade of suspicion of aortic dissection as a possible alternative in presence of eletrocardiographic myocardial ischemic signs. In many medical centers where thrombolitic therapy, antiplatelets receptor blockers, heparin or percutaneous coronary angioplasty is the first line therapy for ACS the outcome may be catastrophic in situation such as aortic dissection.

## Introduction

Aortic dissection is a rare but catastrophic disorder that affects the aorta. It has a frequency of about 2000 cases/year in US. The mortality rate for untreated patients is reported to be 1 - 2 % /hour during the first 48 hours after development of symptoms; the range of manifestation is extensive and mis-diagnosis is common [1].

We describe a case of a 54 years old man in whom an initial diagnosis of acute coronary syndrome (ACS) revealed to be finally an acute aortic dissection.

This case report stresses the importance to maintain a high grade of suspicion of aortic dissection as a possible alternative in presence of eletrocardiographic myocardial ischemic signs.

In many medical centers where thrombolitic therapy, antiplatelets receptor blockers, heparin or percutaneous coronary angioplasty is the first line therapy for ACS the outcome may be catastrophic in situation such as aortic dissection.

## Case Report

**Figure 1:**
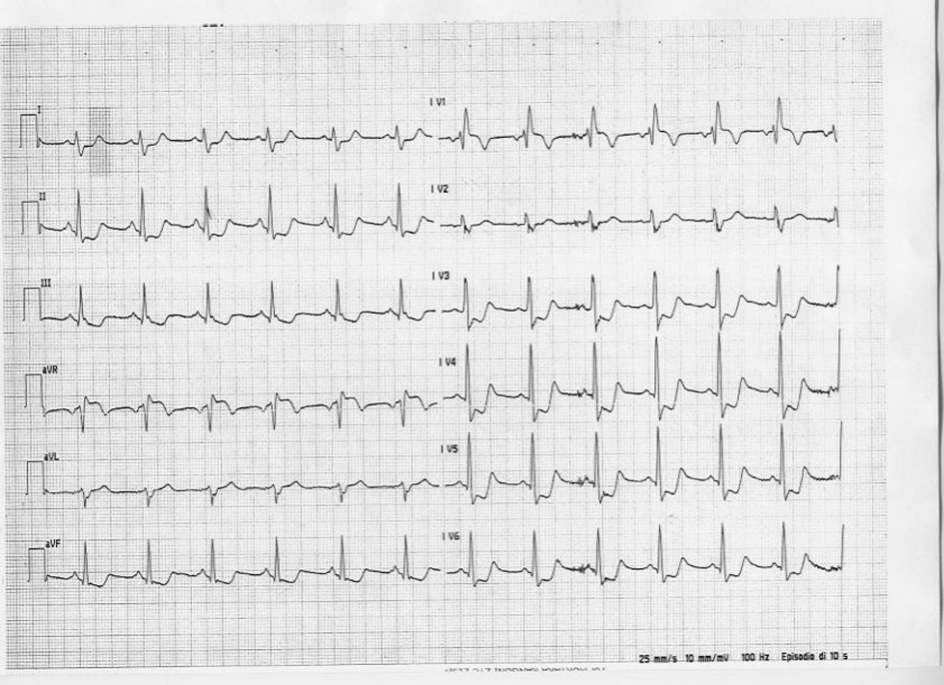
Admission electrocardiogram.

A 54-year-old man, presented with sudden anterior chest pain, irradiating to the jaw, followed by profuse sweating, and vomiting. He was assisted 1 h after the onset of the symptoms. The patient had a history of arterial systemic hypertension, but no family history of coronary heart disease and no other cardiovascular risk factors. On admission, the blood pressure was 150/60 mmHg and the heart rate was 74 bpm. Cardiac examination revealed a diastolic murmur. The electrocardiogram obtained on admission (Fig. 1) revealed a ST segment depression in precordial V3-V6 leads, inferior D2, D3, aVF leads and a ST elevation in aVR lead. Chest x ray did not show mediastinum enlargement. He was firstly diagnosed to have an acute massive anterior infarction and haemodynamic procedure was urgently scheduled.

**Figure 2:**
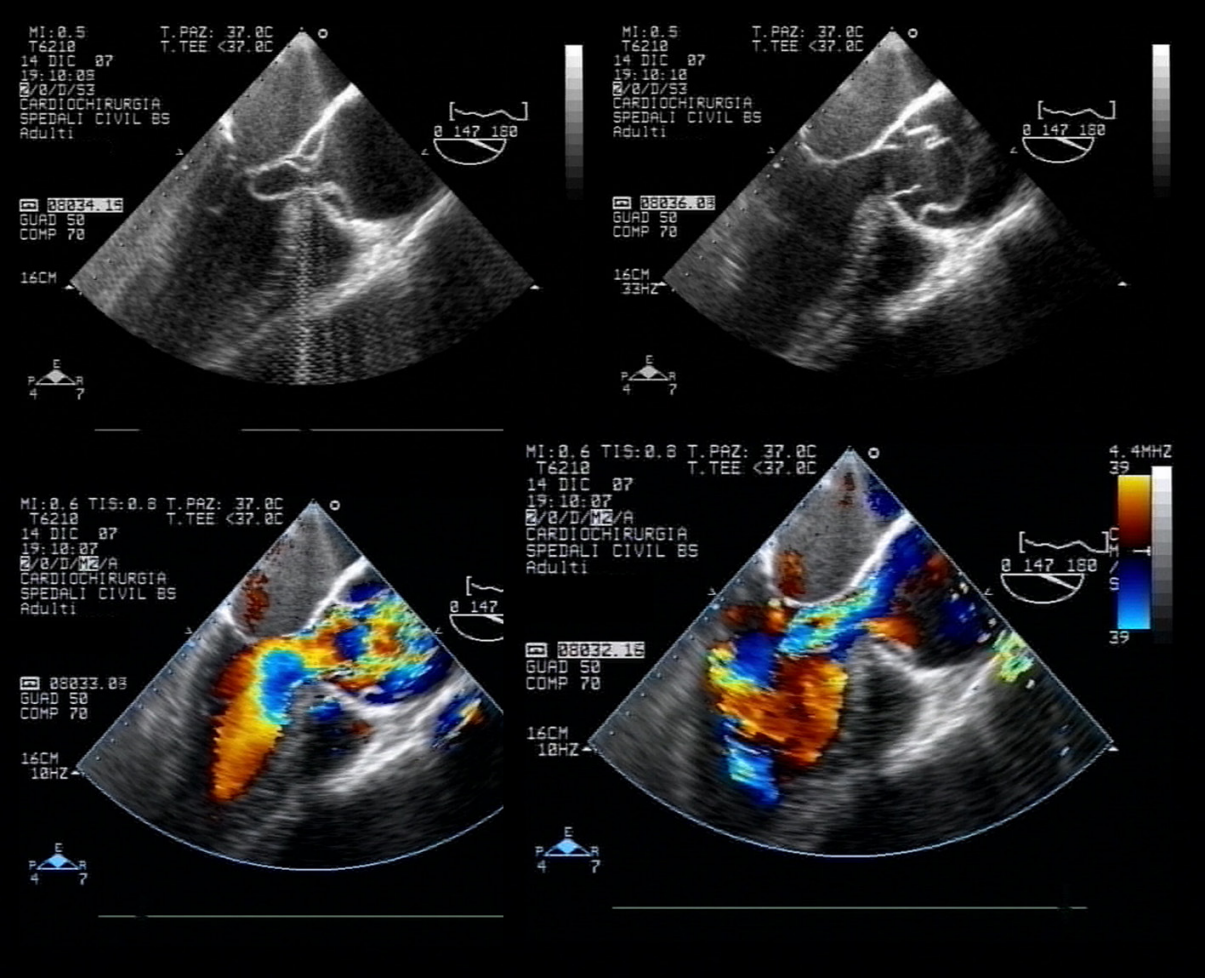
Transesophageal echocardiogram images of aortic dissection.

The patient was also submitted to a bedside 2- dimensional transthoracic echocardiogram that not revealed significant segmental kinesis alterations, but the presence of an extremely mobile undulating intimal flap within a dilated ascending aortic lumen, and severe aortic regurgitation. A following transesophageal echocardiogram confirmed the diagnosis of a Stanford Type A aortic dissection with a thick intimal flap, separating the true and false lumen, intermittently occluding both coronary ostii and floating into aortic valve plane (Fig. 2). The entry of the dissection was 1 - 2 cm above the commissure of the aortic valve and extended to half of ascending aorta. After an initial treatment with intravenous nitrates and beta blockers, the patient underwent an emergency complex aortic surgery consisting of aortic root replacement with a mechanical aortic valve conduit.

The patient had an uncomplicated post-operative course without myocardial residual damage and he resumed a normal activity after hospital discharge.

## Discussion

Aortic dissection is a life-threatening cardiovascular event [2]. Early mortality is as high as 1% per hour if untreated, but survival may be significantly improved by the timely institution of appropriate medical and/or surgical therapy [3].

Patients with suspected thoracic aortic dissection require early and accurate diagnosis. Involvement of the ascending aorta (type-A dissection) is moreover critical, due to its proximity to the heart [4, 5]. About 20% of patients with type A aortic dissection have also ECG evidence of acute ischaemia or AMI, and may go unsuspected or undiagnosed. The right coronary artery is more involved than the left. In the International Registry of Acute Aortic Dissection [6], a normal electrocardiogram was present in less than one third of patients and signs of myocardial infarction (new Q waves or ST-segment elevation) were detected in 4.8% of patients with type A and in 0.7% of patients (only one case) with type B aortic dissection. Accordingly, this case emphasizes the opportunity to consider dissection of the descending aorta even in the presence of chest pain with ischaemic ECG changes. We reported a case of type-A dissection, in which the intimal flap and the false lumen involved right and left main coronary ostium with electrocardiographic features suggestive of non-ST myocardial infarction. Emergent primary coronary angioplasty and eventually an intra-aortic balloon pumping has been recommended for the management of patients with acute myocardial infarction and critical main trunk disease, as the first line reperfusion therapy. However, a correct diagnosis in patient with ACS signs and underlying aortic dissection is crucial before invasive haemodynamic procedures settlement [7]. A rapid non invasive instrumental evaluation such as TT/TE echocardiogram appear to be always essential as a first screening in patients presenting with acute chest pain symptoms.

In our case, we believe that the chance of survival of this patient would have been very low if any delay or instrumental misdiagnosis (echocardiographic postponing) was occurred.

Aortic dissection should be ruled out whenever a patient presents with EKG signs suggestive of acute myocardial infarction at the emergency department. A bedside physical examination and a chest x-ray study are usually insufficient modalities to evidence the presence an aortic dissection which need be confirmed by more sophisticated means such as transthoracic and transesophageal echocardiography or helical computed tomography (CT) or magnetic resonance imaging (MRI).

The emergency physician should always keep a high index of suspicions even in presence of severe EKG alterations significant for myocardial ischemia. Undoubtedly, an immediate correct diagnosis and emergent surgery can be lifesaving.
